# User generated content for enhanced professional productions: a mobile application for content contributors and a study on the factors influencing their satisfaction and loyalty

**DOI:** 10.1007/s11042-021-11381-2

**Published:** 2021-08-24

**Authors:** Stavroula Ntoa, George Margetis, Fiona Rivera, Michael Evans, Ilia Adami, Georgios Mathioudakis, Rajitha Weerakkody, George Metaxakis, Ioannis Markopoulos, Marta Mrak, Constantine Stephanidis

**Affiliations:** 1 Foundation for Research and Technology – Hellas (FORTH), Institute of Computer Science (ICS), 70013 Heraklion, Crete Greece; 2 BBC Research and Development, London, UK; 3 Forthnet S. A, Athens, Greece; 4 Department of Computer Science, University of Crete, Heraklion, Greece

**Keywords:** User generated content, Media sharing, Professional broadcasting productions, Field study, User satisfaction, Customer loyalty

## Abstract

Motivated by a combination of social media, technological evolution, as well as new habits and preferences of TV content consumers, there is an increasing demand for enhancement of professional productions with user generated content. Studies have explored the potential and feasibility of this approach, indicating that footage from non-professionals can be effectively used to enrich the viewing experience. However, an important concern is whether such efforts are appealing to potential contributors, and what can actually impact their satisfaction and loyalty. Aiming to investigate these factors, this paper presents a mobile application for content contributors and a study involving 38 attendees of live events, using the application in the field. The events were hosted in two different countries, and transmitted by two well-known broadcasters. The results suggest that age, gender, technological expertise, and overall sharing attitude do not affect the satisfaction and loyalty of contributors. The differentiating factors, however, are the filming confidence and expertise of contributors, as well as the Wi-Fi/4G connectivity on-site. Implications of these findings are discussed and recommendations for similar endeavors are provided.

## Introduction

It wasn’t very long ago that channel-hopping was the only means for TV viewers to control the content they would consume. The technological evolutions of the new millennium, brought about by the proliferation of mobile devices and social media, have shaped an entirely new landscape for enriched media experiences. Television is still a powerful medium, playing an important role in everyday life. However, it has to evolve in order to address people’s new viewing habits [26]: watching television content time-shifted, viewing content on mobile devices, and employing multiple screens [24]. At the same time, viewers have exhibited their preference towards more social TV experiences, such as real-time online discussions with friends, sharing and recommendation of video material according to social network statistics, and direct connectivity with relevant social media [3]. At the same time, there are cases when world events (*e.g.* a natural disaster, a pandemic such as the Covid-19) intensify the need for video captured without the extensive involvement of professionals, as a response to the increasing demand of the general public to be entertained, informed and educated by media.

Inspired by new these trends, capitalizing on the power of social media and the high resolution cameras with which mobile devices are now equipped, several efforts have suggested accompanying mainstream productions with user generated content, so that viewers can acquire a feeling of “being there” [32, 33]. In principal, the idea is enticing and has the potential to leverage interactive media experiences, fostering viewers’ feeling of presence and connectivity [10, 16]. In addition, professional producers have expressed the desire to integrate contributions from the audience alongside professional coverage of live events, in order to convey alternative viewpoints, authenticity and variety [16]. However, when applied in practice, several challenges have to be addressed, so that the User-Generated Content (UGC) is of appropriate quality. Concerns relate to the willingness of spectators to capture and share media; the quality, appropriateness and suitability of the content; the video resolution and audio quality of the filmed video; and intellectual property rights (IPRs) [9].

The COGNITUS platform proposes an integrated solution for interactive media experiences featuring UGC. Content from users can be uploaded to the COGNITUS platform through a dedicated mobile application in response to calls from professional broadcasters for contributions. IPR is typically handled by event organisers and performers^1^,^2^, but in the case of COGNITUS, it is also addressed through terms and conditions transparent to users within the mobile app. The submitted UGC is automatically transformed to ultra-high resolution, through the platform’s backend video processing mechanisms, and the enhanced UGC content is served to the end-users of the platform. Professionals can review all the submitted content and select contributions to integrate into their mainstream broadcasting, or to accompany it on viewers’ secondary screens (*e.g.* tablet devices) and on set-top boxes (STBs). Suitability and appropriateness of content, as well as the preservation of IPRs, is assured via content reviewing by the professionals in addition to a “report content” functionality available for users, which acts as a safety feature. An important concern when deploying solutions that are inherently based on user content, is how to ensure that content contributors will not only be motivated, but will actually contribute content. Are there any subjective or objective factors that influence the attitude of contributors, and if yes how can UGC-based endeavors account for them?

This paper introduces the platform’s mobile application, the medium for submitting UGC, and reports on its empirical field evaluation with real users, during events that were hosted in two different countries: sports events (football games) in Greece and cultural festival events in UK. In conducting this ‘in-the-wild’ investigation, the core research question focused on: what are the demographic, behavioral, attitudinal and other contextual factors that influence a UGC contributor’s satisfaction and loyalty, to a platform of this kind? As such, the following hypotheses were explored in the context of this study:

- H1: Event attendees will be positive towards using such an app for recording and contributing through it content to a UGC-enhanced media platform.
- H2: Younger individuals are more likely to be more satisfied with the app and become loyal contributors.
- H3: Individuals familiar with smartphones are more likely to be more satisfied with the app and become loyal contributors.
- H4: Individuals who are adept at filming are more likely to be more satisfied with the app and become loyal contributors.
- H5: Individuals who regularly share content in social media are more likely to be more satisfied with the app and become loyal contributors.

The remainder of this paper is structured as follows. Section 2 reviews related literature on UGC for interactive media. Section 3 provides a description of the COGNITUS mobile application. Section 4 describes the evaluation methodology and presents the evaluation results. Section 5 discusses the findings, while Section 6 concludes the paper and provides directions for future work.

## User-generated content for interactive media

When considering the enhancement of TV broadcasting experiences with crowdsourced content, principal points of concern are the suitability of the content in terms of quantity (*i.e.* if adequate coverage of the event can be achieved) and the content quality (*i.e.* if UGC-enhanced productions can produce a better viewing experience). Research efforts have studied content contributions by users in different event types. In the context of a marathon event, a trial study exploring the potential of spectators for creating footage, reported that a rather small number of spectators can capture a significant amount of video and concurrently index it with tags, also exhibiting good temporal coverage of the event, but a more patchy geographical coverage [12]. Findings from field trials in sports events of the *CoStream* system, a system that supports mobile live sharing of UGC video, suggested that real time sharing of different perspectives from an event can create new experiences for event spectators themselves, as the media sharing activity enhances and intertwines with the overall event [6]. Similar findings were reported by an earlier study, examining how spectators’ experiences of a world rally championship were re-shaped when they used mobile phones for recording multimedia from the event [14]. With regard to the feeling of “being there” when watching live events from home, a recent study identified that the experience bears a complex relationship with the actual experience of being at the festival [32]. Factors that were reported to have an impact on the feeling of “being there” included objective technical issues, such as sound and image quality, as well as each individual’s knowledge and expectations of the event. *Bootlegger*, a prototype system for creating multi-camera films of live music events [29], has shown the feasibility and potential value of lowering the barrier to entry to professional-type media production workflows, to non-professional users, including fandoms and campaigning communities (*e.g. * [5]).

A promising research area taking advantage of UGC footage of events has been identified as mixing UGC content with content provided by the event organizers [15]. So far, to our knowledge, only few such platforms have been reported. *WanderCouch* is a smartTV application that enables users to experience the events from a distance [33]. *WanderCouch* focuses on music festivals, and includes a variety of features such as blending professional audiovisual footage with UGC, offering glimpses of the festival’s side events, and virtually navigating the festival spatial area. The evaluation of the system was carried out in a laboratory set-up aiming to assess the smartTV application and the viewing experience. Results suggested that the approach has a great potential to improve traditional TV broadcasts, however real-life experiences cannot be replaced as they are much more complex. Another platform reported in the literature is *ICoSOLE*, which aims to constitute an integrated approach for combining content from professional and consumer capture devices, including mobile (and moving) sensors, based on metadata and content analysis [1]. However, the work seems to be currently in the research and development phase, and no evaluation results are reported.

The COGNITUS platform builds upon the potential of combining UGC with professional productions, and supports any type of events. This includes events where people are physically present (such as a festival or sports game), or events of interest to producers to gather personal stories and feedback in response to world events (*e.g.* people reporting on their Covid-19 experiences). Taking advantage of the knowledge and expertise of professionals, producers are assigned an active role in selecting and promoting the broadcasting of UGC that is topical (*e.g.* content that complements their footage, exclusive, or engaging content). This paper describes the mobile application that event spectators use to upload footage and reports results from its evaluation in field trials at two different types of events in two different countries: an international arts street festival and live sports games. Evaluation in the field aimed to assess not only the experience of uploading footage, but also how loyal the customers of such an application would be, which is a crucial parameter for any approach that heavily relies on UGC. A unique contribution of this work is also the exploration of the various factors that potentially influence the overall satisfaction and loyalty of content contributors, including age, gender, familiarity with the technology involved (*i.e.* smartphones), as well as filming skills and confidence.

## The COGNITUS mobile application

The COGNITUS platform targets capturing, aggregating, enhancing and distributing UGC for professional broadcast use. The platform includes a number of services, developed in a user-centered approach, taking into account requirements and feedback from production professionals of the broadcasters involved in the project, namely BBC and NOVA, the largest pay-tv platform in Greece. This paper focuses on the description and evaluation of the mobile application that is used by contributors to capture and upload video footage, which is a core activity for the proliferation of the COGNITUS platform and community.

The main objective of the mobile application is to enable users to record and upload footage while an event is going on or asynchronously (after an event). When footage is uploaded via the mobile app to the COGNITUS platform, the application triggers a series of server-side processing components, for upgrading its quality and audio-visual characteristics (*e.g.* spatial and temporal resolution) [7, 30]. As a result, the platform undertakes the task of delivering high quality videos, regardless of the quality of the initially contributed videos. The upgraded content can then be delivered back to the end-users via the mobile application, the COGNITUS platform STB and its companion application. In addition to viewing the enhanced versions of their uploaded videos, users can also view videos uploaded and shared by other users for any event supported by the COGNITUS platform. By selecting a specific open call or past event, users can browse through all the relevant clips whether UGC or professional productions. In a nutshell, through the mobile app users can: browse and search for events, upload their own footage to an event, as well as view content from events. In addition, the application also includes social and user reward features, namely followers and badges, to motivate loyalty and content contributions.

**Fig. 1:**
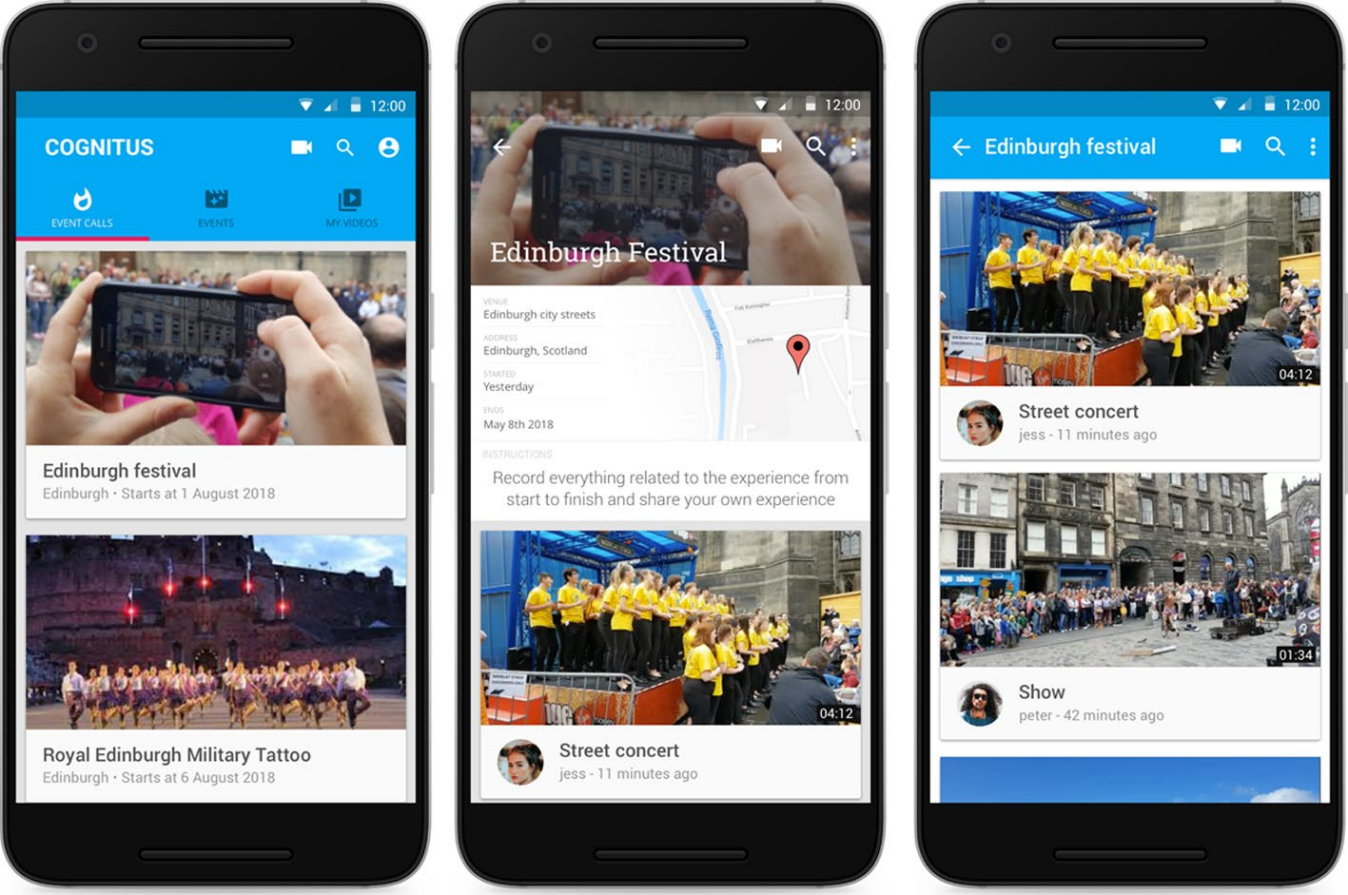
Mobile application screenshots (left) home screen (middle) event screen (right) event screen scrolled down

The home screen (Fig. 1 left) of the application, titled “Event Calls”, acts as a gateway for users to discover calls for contributions, based on their current location and temporal relevance (*e.g.* currently active events). Each event listing appearing on the home screen includes some basic and distinct information such as, the name, the venue, a corresponding thumbnail and the start date of the event. Additionally, the upper part of the screen includes a map that displays the geographical location of the event. The home screen features a lazy-loading mechanism, which delays fetching of content until it is needed, as the user scrolls down the page to the bottom, up to the point where no further qualifying events are to be shown. Once the user selects a call for contribution, the event details screen Fig. 1 middle and right), appears, displaying event-related information, details of the call (i.e., what the producers are looking for), as well as the currently associated publicly available (video) content items. The content list includes a thumbnail of the video, the title provided by the contributor, the username of the contributor, the upload time and its duration.

Through the “Events” main menu option, users can view videos contributed and shared to previous events. The events page adopts the rationale of the home page, presenting for each event a card which features a thumbnail, the event title, the event location, as well as the date of the most recent content contribution. Alternatively, users can search for an event, through the search option, where they can type the event name, event location, or contributor name they are looking for.

**Fig. 2:**
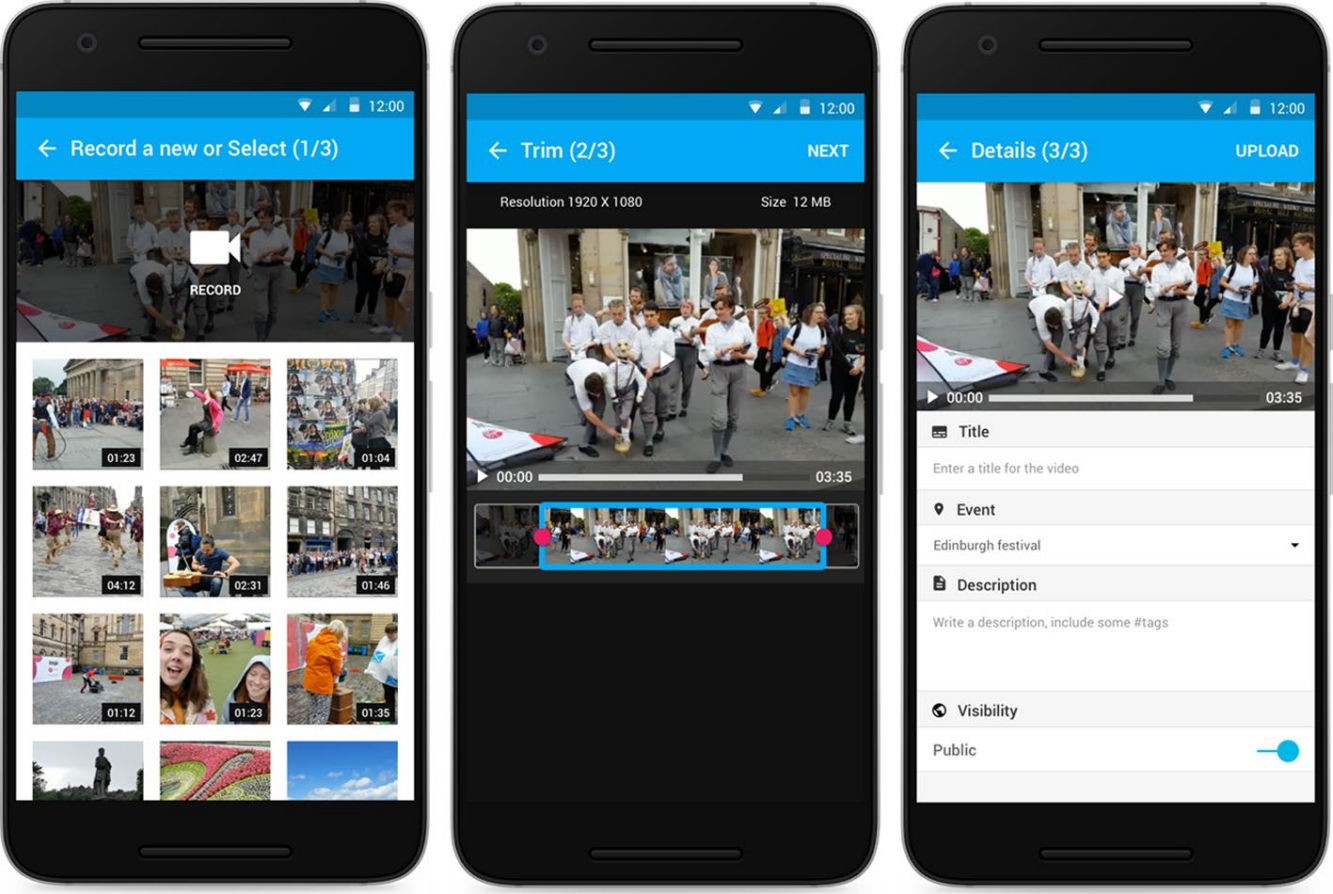
Footage uploading (left) video selection (middle) video editing (right) video details

In order to upload new footage users can select the corresponding camera icon located at the header section of the application. Via the capture screen (Fig. 2 left), a new recording can be initiated or a pre-recorded clip that is already stored in the device can be uploaded. The application also supports some basic editing functionality (Fig. 2 middle) on the content at hand. More specifically, the user can trim the video by changing the start and end times using a slider. The approximate size of the video (in megabytes), which is displayed on the top left of the screen, is immediately updated each time the user modifies the total duration of the video. In addition, a preview of the resulting clip is available via the main area of the screen. The final step (Fig. 2 right) of the upload process involves enriching the new content with information (title, description, event, content visibility). The title of the content is a required field since it will be displayed in the content lists across the users’ devices. The description field facilitates the semantic tagging of the content. Using this field, the user can optionally provide free text and hashtags. The system will suggest relevant hashtags, which have been generated by back-end semantic enrichment components [23, 31]. This functionality is triggered when the user inserts a hash character (“#”) in the description field. At that time, a suggestion dropdown menu appears including listing-related tags. The event that this content will be associated with, is pre-selected according to the location of the user. However, the user is able to modify that selection and associate the content to a different event, listed in the events dropdown menu. The application offers the option for postponing the upload for later, a functionality that can be valuable when the network availability is poor or when Wi-Fi is not available, which would induce charges from the network provider.

**Fig. 3:**
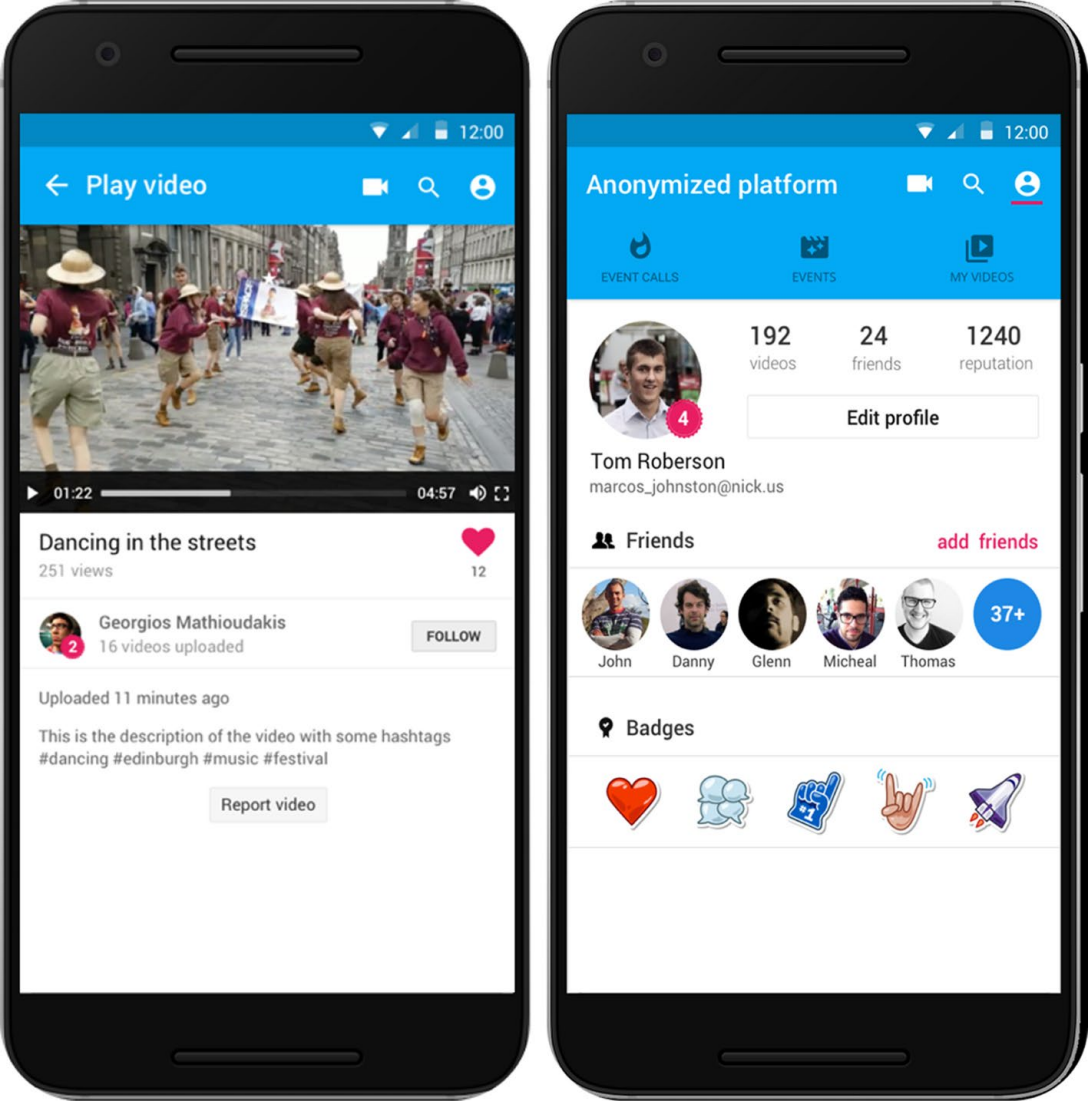
(left) play video screen (right) user profile screen

Besides uploading content, users can also view videos uploaded from other users of the platform. The videos can be previewed using the embedded video player at the upper part of the screen (Fig. 3 left). Moreover, through this screen the user can ‘like’ the content or ‘follow’ the content creator. Finally, a user can report a content if it does not comply with the platform’s terms of use and privacy policy. The reported content is moderated by the appointed producers or the platform administrators and stops being available if they discard it. Furthermore, users can view the profile of a content contributor, as well as their own user profile, featuring the number of uploaded videos, followers and awarded badges (Fig. 3 right).

## Evaluation

Following an iterative design process, the COGNITUS platform has undergone a number of evaluation stages, including functionality testing, formative expert-based evaluation, as well as field trials and user-based evaluations. The evaluation reported in this paper focuses on the final mobile application, after improvements were made during previous evaluation rounds. The evaluation was carried out in the context of real-world events in two different countries. More specifically, it was evaluated during an international arts festival in UK, broadcasted by BBC, and three football match games in Greece, broadcasted by NOVA.

### Methodology

Field (in-situ or in-the-wild) studies are criticized for the inherent cost they bring about and the difficulty in carrying them out in terms of administration and effort, when compared to laboratory evaluations [19] or simulated real world environments [17]. Nevertheless, it is widely acknowledged that although a field study may not identify many new problems when compared to other methods, it is the only type of evaluation that can study user behavior, social comfort in use, and “system in the world” issues, especially when it comes to mobile applications [8, 18, 25]. In particular, with regard to user behaviors, it has been noted that users behave more negatively in the field than in the laboratory, while some behaviors can only be observed in the field [8].

Along these lines, the study of the COGNITUS mobile application presented here, mainly aimed at exploring user behaviors in the field (under real live event conditions), focusing on their satisfaction and loyalty. The primary focus of this study was not to identify usability problems, given this was addressed during earlier evaluation iterations, and the identified usability problems had already been resolved prior to the field trails. Two questionnaires were employed in order to assess the overall satisfaction and potential loyalty of content contributors: the System Usability Scale (SUS) and the Net Promoter Score (NPS). SUS is a simple ten-item questionnaire for measuring user satisfaction [2], which has been extensively studied and reported to be a valid tool for measuring user satisfaction with an interactive system [20]. NPS is a single question tool to measure customer loyalty by asking users to indicate on a scale from 0 to 10 how likely it is that they would recommend the product or service to a friend [27]. According to their score, respondents can be classified as: detractors (scores 0–6) who may not purchase again and could spread negative word of mouth if something is not done to improve their experience; passives (scores 7–8) who are typically satisfied—but not to the extent that they become loyal—and who will spread neither negative nor positive word of mouth; and promoters (scores 9–10) who are considered the most loyal and enthusiastic customers that will spread good word of mouth. Given the NPS range of − 100 to + 100, a “positive” score or NPS above 0 is considered “good”, + 50 is “Excellent,” and above 70 is considered “world class”. Research on NPS has yielded controversial results, mainly stemming from the fact that by just asking one question, valuable information that could explain participants’ responses is lost [28, 34]. In the current study, this limitation is resolved by combining NPS with SUS and open-ended questions (see below), instead of using it as a single instrument for measuring customer loyalty or user satisfaction.

In order to add more depth to the analysis, two more sections were added to the final questionnaire so as to elicit demographic information and relevant background expertise, as well as an open-ended questions part that would allow participants to express their thoughts. As a result, the final questionnaire was structured with the following sections: (i) demographics (*i.e.* age range, gender, occupation, filming expertise); (ii) background expertise and attitude (confidence with mobile apps, confidence in the current day’s filming, frequency of attending events such as concerts, festivals, and sports games, frequency of sharing videos on social media, frequency of posting videos with oneself); (iii) SUS; (iv) NPS; (v) open ended questions (favorite features, suggestions for improvements and suggestions for future features, recommendations on types of events suitable for such UGC solutions). Questions of the second section (ii) were given in a 5-point Likert scale format, with answers ranging from 1 to 5.

### Procedure and participants

Spectators were informed of the evaluation and the procedures that would be followed prior to entering the event. Upon expression of interest to participate in the study, more detailed explanations and informed consent forms were given to the study participants to sign. An eligibility criterion for selecting attendees to participate in the survey was to be familiar with using an Android mobile device since the application was available only for Android platforms.

The evaluation facilitators introduced each participant to the aims and objectives of the application and gave them a short period of time to interact with the downloaded application. During the evaluation, participants were allowed to use the application freely at will, without structured tasks or observation. However, in order to ensure some consistency in use, they were asked to use the COGNITUS mobile application to contribute event content at least five times but whenever they wished. They were also asked to respond to the questionnaire before leaving the event. All questions and enquiries of the participants regarding the process, the application, or the questionnaire were addressed before they entered the event.

In total, 38 users participated in the trials, with 22 users (58%) being attendees of sports events and 16 (42%) attendees of festivals. Demographic information of the participants is presented in Table 1 and background expertise information in Table 2. It is noted that 7 sports events participants did not respond to questions regarding their expertise, and therefore the analysis and reporting of background expertise pertains to the remaining 31 questionnaires.

**Table 1:** Participants’ demographic information

Gender	Male	25 (65.79%)
	Female	13 (34.21%)
Age	18–24	5 (13.16%)
	25–34	11 (28.95%)
	35–44	6 (15.79%)
	45–54	12 (31.58%)
	> 55	4 (10.52%)
Filming expertise	None-very little	10 (32.26%)
	Hobbyist—some but primarily for self and small circle of contacts	15 (48.39%)
	Student—have taken courses or currently studying film/photography/video	1 (03.22%)
	Influencer—like to share on social media with a wide circle of followers	5 (16.13%)
	Professional—do it for a living	0 (00.00%)

**Table 2:** Participants’ background expertise

Confidence in	Mobile apps		That day’s filming	
1 (None)	0		0	
2 (Some)	0		0	
3 (Medium)	2 (06.45%)		3 (09.68%)	
4 (Good)	16 (51.61%)		12 (38.71%)	
5 (Very good)	13 (41.94%)		16 (51.61%)	
*Frequency of*	Attending events	Sharing videos from events on social media		Posting videos with oneself on social media
1 (Never)	0	4 (12.90%)		6 (19.35%)
2 (Rarely)	3 (09.68%)	5 (16.13%)		9 (29.03%)
3 (Occasionally)	5 (16.13%)	9 (29.03%)		9 (29.03%)
4 (Often)	14 (45.16%)	10 (32.26%)		4 (12.90%)
5 (Very often)	9 (29.03%)	3 (09.68%)		3 (09.68%)

### Results

Overall, participants contributed a considerable amount of footage, contributing a total of 1,100 videos for the cultural event and 817 videos for the sports event. As a result, festival attendees contributed on average 68 videos each, sports event attendees contributed on average 37 videos each, while the overall video contribution (as calculated from both events) is 50 videos per participant on average. Although the purpose of this study was not to assess the quantity of the contributed videos, this metric is reported as an indication of the validity of the questionnaire responses, highlighting that all participants reported on their experience after having used the application quite extensively. Also, assessing the quality of contributed videos was beyond of the scope of this study, mainly since the COGNITUS back-end undertakes the task of up-scaling videos to high quality.

#### Contributor satisfaction

The SUS questionnaire was used to quantitatively measure satisfaction of contributors using the COGNITUS mobile application. The overall final average SUS score was 76 (95% CI [70.70%, 80.74%])). The overall average is higher than 68 which is considered an average SUS score [21]. The lowest average score is also expected to be greater than average for the general population with 95% confidence (based on the CI-LL). According to the score received and the benchmarking proposed in [21], the application is characterized as acceptable (Grade B), with 95% certainty that the general population would characterize the application at least as acceptable.

**Fig. 4:**
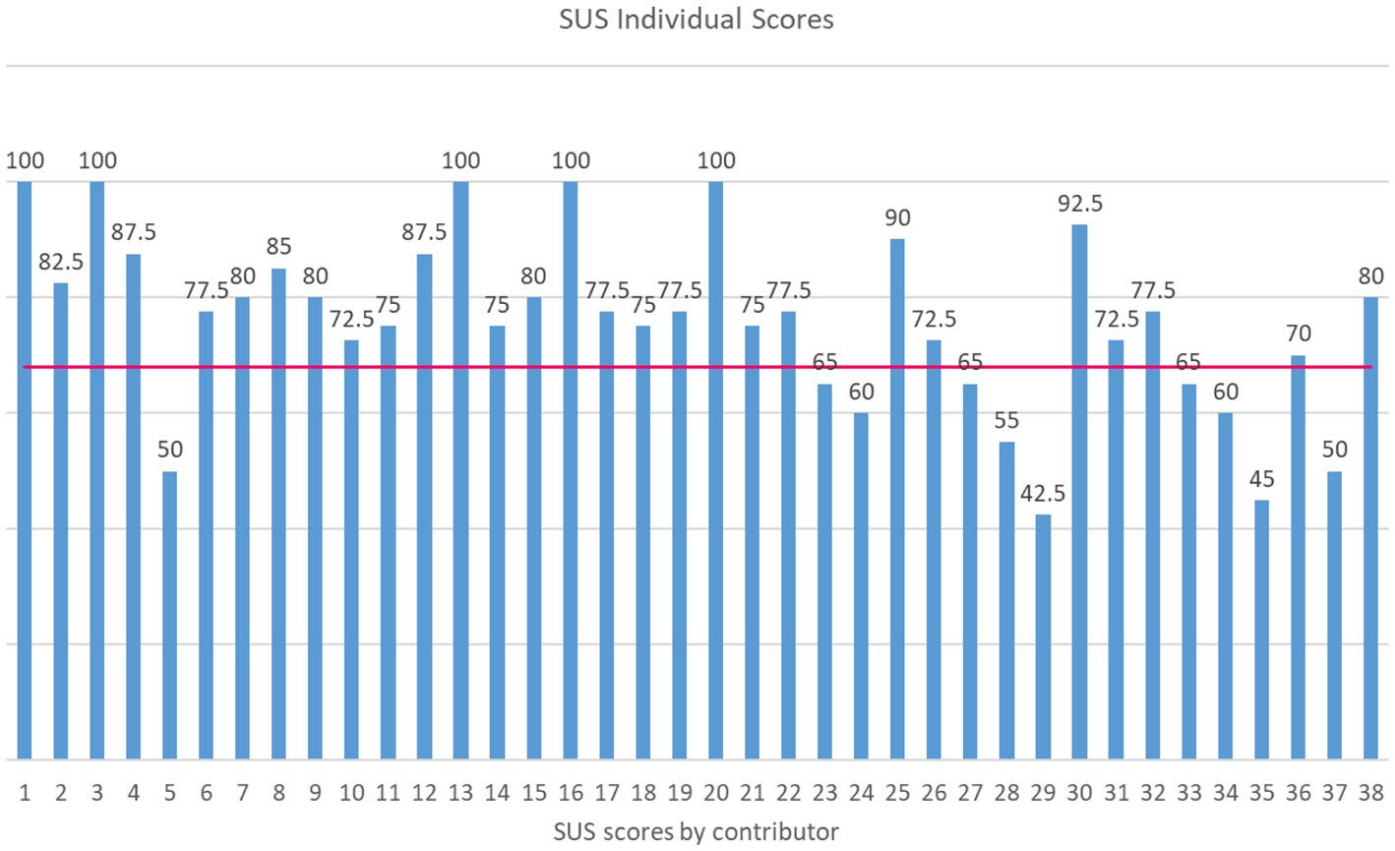
Individual SUS scores. The red line denotes the score threshold of 68

Analysis of the individual SUS responses indicated that 73% of the contributors gave a usability score above the average and 27% gave it a score below (see also Fig. 4). In particular, a considerable portion of participants (26.31%) gave a score higher than 84.1 (Grade A +), while the overall percentage of participants characterizing the application in the grade range of A (Grade A-, A, or A +) is 39.47%. Upon further investigation into the profile of those 10 contributors who gave a lower than average score, it was revealed that 5 had stated “none—very little” experience in filming/video and the other 4 stated that they film videos as a “hobbyist” in the questionnaire. Furthermore, in the question to list three features that they would like to see improved in the application, all 10 of them noted similar issues during the trials, either with the uploading video speed, or the Wi-Fi/4G signal, which caused some user frustration. A potential explanation is that the SUS scores of these users were lower than average due to the frustration and inconvenience caused by their very low experience with filming/video in combination with the connection issues which made video uploading slow and cumbersome. It is also notable that 6 of the 10 participants who gave a low SUS score also gave a detractor NPS (0–6 score), 3 gave a passive NPS (7–9), and only one gave a promoter NPS (9–10 score).

#### Contributor loyalty

**Fig. 5:**
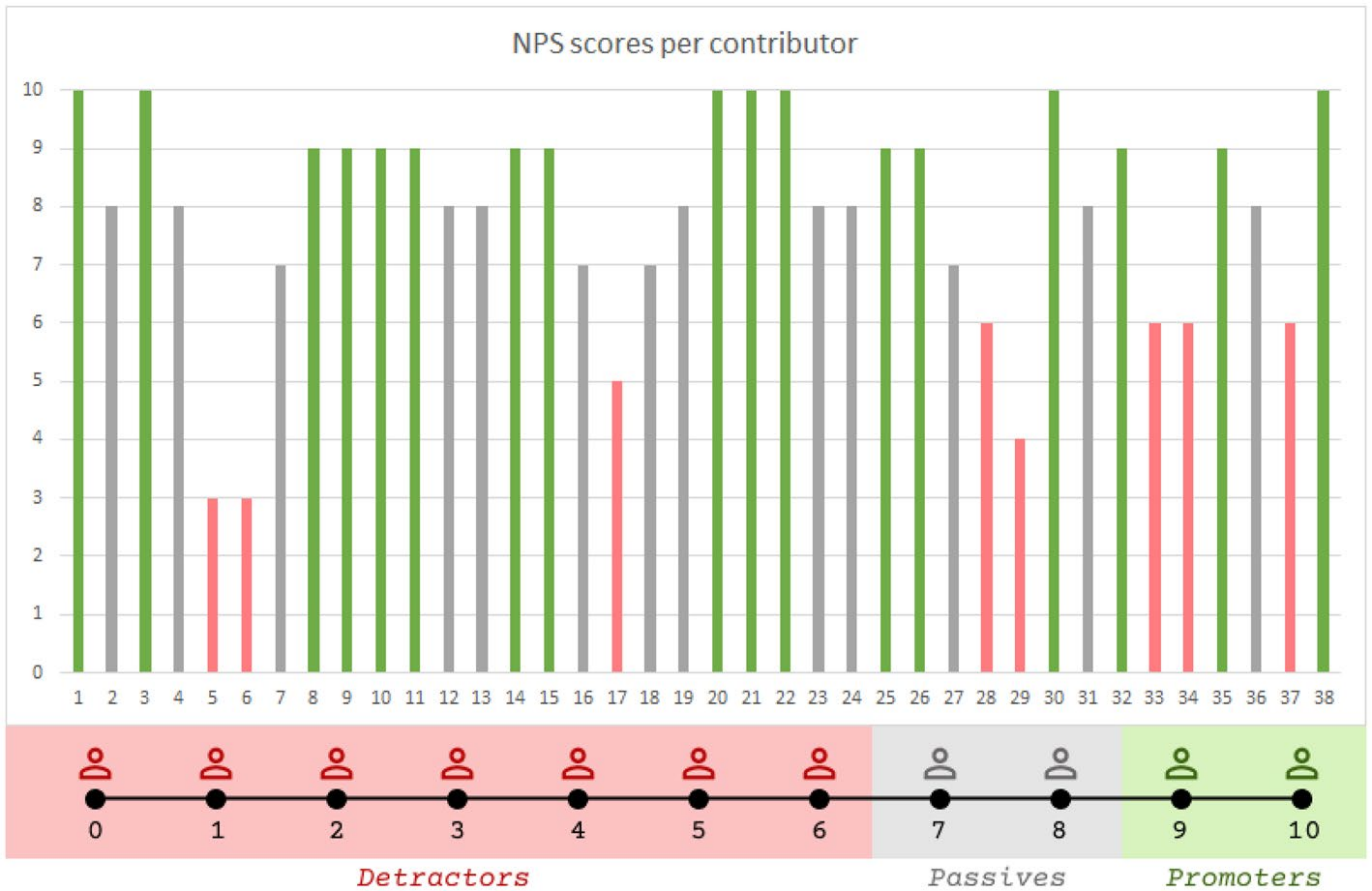
NPS scores per contributor classified to promoters, detractors, and passives

The total NPS for the mobile application was 24%, which is a good score considering that the scores can range from “− 100” to “ + 100” and therefore anything above zero is a positive indication for the product. Figure 5 illustrates the individual NPS contributor scores, highlighting detractors, passives, and promoters. Overall, the average NPS score was 7.86 (95% CI [7.24%, 8.49%])), suggesting that we can conclude with 95% certainty that general population contributors will be passives (both in the worst and best case), thus neither promoting nor demoting the application. Analysis of the individual NPS scores indicated that 44.73% of the participants can be classified as promoters, 34.21% as passives, and 21.02% as detractors.

#### Open-ended questions

Open-ended questions asked participants to: (a) list up to three of their favorite features in the application, (b) list up to three features that they would like to see improved, (c) suggest up to three future features, and (d) suggest event types for which the app would be suitable.

Responses were analyzed manually, following a combination of deductive and inductive coding [11]. In particular, since open ended questions asked participants to list system features, one code for each one of the functions provided by the system was created in the initial predefined codes, following the deductive coding approach. Then, the researchers divided the data into two sets and examined the responses acquired in order to assign one of the predefined codes for the first set. In the cases when the need for assigning a new code was identified, this was added to the set of codes, and all responses were re-examined, following the inductive coding approach. The examination of responses and code assignment was carried out by two individual researchers. The outcomes of the two individual analyses were initially compared for the first data set. In those cases where inconsistencies were observed in the codes assigned, further elaboration followed through discussion between the two researchers. The new coding frame that was produced was used to analyze the second set of responses, following the same consensus building approach.

A feature that received the most mentions as a favored was the video upload functionality (listed by 23 contributors, 60.52%). It was appraised for providing the option to upload at a later time, ease of use in capturing and uploading videos, and video quality enhancement. The feature that received the second most mentions was the ease of use of the application in relation to the design of the interface and the overall offered functionalities (by 22 contributors, 57.89%). The feedback received regarding the ease of use is consistent and supports the high SUS score that the application received. Finally, another favored feature (by 14 contributors, 36.84%) was the categorization of the events into upcoming, running, and nearby, which makes it easy for the users to find the information they are interested in. Other features mentioned as favorites by the contributors, were the use of hashtags, the possibility to follow someone else’s videos, possibility to upload a video at a later time, the overall content quality of the application, and lastly the overall concept of sharing content. Table 3 lists the abovementioned features, and provides the most representative comments for each one.

**Table 3:** Thematic analysis of responses provided for favorite application features

Feature	Comments	# of mentions
Video upload	Fast and direct video upload Possibility to upload at a later time Easy and direct way to capture videos Easy upload facility Recording system was responsive The storage of the videos was practical	23
Overall ease of use	Simple design User-friendly application Simple menus Simple UI Easy to understand UI Easy to understand functionalities Sequence of functionality is sensible	22
Content organization	Nicely aggregated content Easy to see the available, upcoming, and nearby events Grouping of videos per event (which means easy searching in previous events) Possibility to learn about interesting events in sports and culture I can see which events are nearby me, event calls, the categories Easy to see list of available events Being able to categorize Topics—separate genres of filming (i.e. different events) My videos view	14
Overall concept	Very smart concept of sharing content Interesting result Liked showing the game from the stands for the people that were not in the stadium Real time video sharing Liked the social aspect of it, possibility to follow other user’s videos	5
Hashtags	Use of hashtags Ease of use of hashtags Add hashtags Tags for searching	4
Content quality	Good quality of videos Clear videos of performers and public	4
Other	Retry button was good & quick to retry User promotion with badges	2

The most common point of distress for participants was the connection speed (via 4G or Wi-Fi), which delayed their upload and created frustration (pointed out by 14 users, 36.84%). Features that more than one participant would like to see improved included: better handling of the device orientation regarding the video thumbnails (2 users, 5.26%), simultaneous uploading of multiple videos (2 users, 5.26%), indication of upload status (3 users, 7.89%), and enhanced interaction between the users (3 users, 7.89%). The last three suggestions were also replicated as ideas for features to include in the future. Other suggestions for future features encompassed the option for uploading and sharing photos besides videos, options for sharing to social media, selection of the video thumbnail, and higher visibility of top contributors. In particular, the suggestions received were classified into six main categories, namely video upload, content organizations, hashtags, social features of the application, video utilities, and photos (Table 4).

**Table 4:** Classification of suggested features

Feature	Comments
Video upload	Faster video upload, show upload status (%), mass video uploading mechanism, indication of which videos have already been uploaded, indication of where videos are stored before uploading, message that no connection is required, allowing for definition of thumbnail for videos
Content organization	Sorting or searching of videos of others who are uploading in the event (e.g. in alphabetic order or by user)
Hashtags	Additional tag suggestions (i.e. more hashtags) and more emphasis on connection of hashtags which connect the UGC opening in social media
Social features	Allow more interaction between users of the application, outreach to other social media, it would be nice to see other people who are at the same event
Video utilities	Video settings on the screen (i.e., zooming, video filters, effects, allow rotation of the vertical videos, etc.), more video description options to choose from (e.g. funny videos, etc.)
Photos	Option for uploading and sharing photos

Finally, in the last question, regarding types of events for which such an application would be suitable, participants indicated the following event types:

- Sports events (e.g. basketball, football, track and field, races. etc.) of local, national and international interest (e.g. the Olympics, world championships, etc.)
- Public city events (i.e., parades and marches, public concerts, street celebrations, etc.)
- Festivals of all types (e.g. music, food, etc.)
- Concerts
- Fashion events
- Theater performances
- Award shows
- Interviews
- Accidents to film and record quickly, troublesome behavior to witness
- Live news reporting, live investigative reporting
- Mass public aggregation events like protests
- You’ve been framed moments

It is interesting that participants’ responses were not constrained to official events, but also highlighted the possibility of such an application to be used for live daily life events, as well as for news and investigative reporting.

#### Effect of contributor’s characteristics and expertise on satisfaction and loyalty

Additional statistical analysis was carried out to explore the effect of the various characteristics and expertise of contributors on user satisfaction and loyalty. In particular, t-tests were used to explore the effect of gender on user satisfaction (t(38) = −0.18, *p* = 0.86) and loyalty (t(38) = − 1.03, *p* = 0.3), without identifying any statistically significant effect. The effect of each one of the other user-reported attributes on user satisfaction and loyalty was explored with one way ANOVAs. In particular, the following effects were explored:

- Effect of participant’s age on satisfaction with the mobile application (SUS score).
- Effect of participant’s age on loyalty (NPS score).
- Effect of participant’s filming expertise on satisfaction with the mobile application (SUS score).
- Effect of participant’s filming expertise on loyalty (NPS score).
- Effect of participant’s confidence in using mobile applications on satisfaction with the mobile application (SUS score).
- Effect of participant’s confidence in using mobile applications on loyalty (NPS score).
- Effect of participant’s confidence in that day’s filming on satisfaction with the mobile application (SUS score).
- Effect of participant’s confidence in that day’s filming on loyalty (NPS score).
- Effect of participant’s frequency of attending events on satisfaction with the mobile application (SUS score).
- Effect of participant’s frequency of attending events on loyalty (NPS score).
- Effect of participant’s frequency of sharing videos from events in social media on satisfaction with the mobile application (SUS score).
- Effect of participant’s frequency of sharing videos from events on loyalty (NPS score).
- Effect of participant’s frequency of sharing videos with oneself from events in social media on satisfaction with the mobile application (SUS score).
- Effect of participant’s frequency of sharing videos with oneself on loyalty (NPS score).

The results of this analysis are presented in Table 5.

**Table 5:** Effect of user-reported attributes on satisfaction and loyalty

User attribute	Satisfaction	Loyalty
age	F(5,38) = 0.10, p = .98	F(5,38) = 0.61, p = .65
filming expertise	F(4,31) = 1.02, p = .38	F(4,31) = 1.63, p = .20
using mobile applications (conf.)	F(3,31) = 1.26, p = .29	F(3,31) = 1.95, p = .16
that day’s filming (conf.)	F(3,31) = 3.13, p = .06	F(3,31) = 4.04, p = .03*
attending events (freq.)	F(4,31) = 0.67, p = .57	F(4,31) = 1.28, p = .30
sharing videos from events in social media (freq.)	F(5,31) = 1.14, p = .35	F(5,31) = 0.43, p = .77
sharing videos with oneself in social media (freq.)	F(5,31) = 1.30, p = .29	F(5,31) = 0.98, p = .46

This analysis indicated that only one self-reported attribute—confidence in that day’s filming—had a statistically important effect on users’ loyalty (*p* < 0.05) and a considerably close to important effect (*p* = 0.06) on their satisfaction. Looking at the individual scores, it turns out that all users who reported medium filming confidence in their filming combined with limited to no expertise, gave rather low SUS (below 65) and NPS scores (detractors).

Finally, SUS and NPS scores were studied for statistically important differences with regard to the event type, through two-sample t-tests. Results indicate that a statistically significant difference existed in contributors’ satisfaction between the two events (t(38) = 3.72, *p* = 0.0006), but not in their potential loyalty (t(38) = 0.49, *p* = 0.62). Nevertheless, with regard to loyalty, further elaboration on classification of respondents to promoters, detractors, and passives, revealed that in the case of festival events the percentage of detractors (31.25%) is double the size of the percentage of detractors in sports events (14%). This observation verifies that participants’ dissatisfaction lead to a higher percentage of individuals who would spread a negative word of mouth. Further exploration of the responses of contributors from each event revealed that individuals who did not feel confident in their filming were all attendees of festival events. In addition, 9 out of the 16 festival attendees, reported issues with the uploading video speed or the Wi-Fi/4G signal, which has already been identified as a factor that had an impact on their SUS scores. In conclusion, the statistically important difference in the satisfaction of contributors between the two events is explained as a result of the Wi-Fi/4G signal connection issues and the perhaps higher self-expectations for filming in public that contributors exhibited in the festival events.

## Discussion

Five main hypotheses were explored in the contest of this study, which aimed to explore if event spectators would be likely to use an application for contributing content towards UGC interactive media experiences and identify motivating factors for sustained content contributions. In this section, all five hypotheses are discussed, and the pertinent findings are summarized.

### H1

Event attendees will be positive towards using such an app for recording and contributing through it content to a UGC-enhanced media platform

This hypothesis was explored mainly by analyzing the contributor satisfaction and loyalty scores, that is SUS and NPS scores, but also through participants’ responses to the open ended questions. The overall user satisfaction score was good, higher than the average SUS score, with the majority of respondents (73%) giving a quite high score, while a considerable proportion of respondents (39.47%) gave an overall score in the range of grade A. Customer loyalty scores indicated that a considerable proportion of participants (44.73%) could be classified as promoters, a percentage that closely corresponds to those who characterized the application in the class of grade A. The overall NPS score for the application was 24%, which is a positive indication for a product, considering that the overall score range is from “− 100%” to “ + 100%”. At the same time, it clearly highlights that there is room for improvements. Analysis of participants’ responses to the open-ended questions identified as a main point of participants’ dissatisfaction the connection speed at the event location, which delayed their upload and as such constituted the core task of the evaluation cumbersome to complete. Furthermore, analysis of participants’ responses to the other open ended questions identified that they appraised the overall concept and ease of use, and identified a breadth of events and occasions for which such an application would be suitable. Based on the above, it can be concluded that event attendees are generally positive for using such an app and for contributing UGC to a corresponding platform, and as such, H1 is confirmed.

### H2

Younger individuals are more likely to be more satisfied with the app and become loyal contributors.

This hypothesis was explored through carrying out statistical analysis to identify the effect of age on user satisfaction (SUS scores) and loyalty (NPS scores), without however finding out any statistically important effect. The role of age and gender in sharing attitudes in social media has been extensively discussed in literature. A considerable number of studies report differences in sharing attitudes between genders and ages (*e.g. * [13, 22]*.*). The current study, being more focused on sharing videos from events in a dedicated platform that is used to enrich professional productions with UGC, did not conclude to any similar findings regarding any effect of age and gender on satisfaction and loyalty. As a result, H2 is rejected, and therefore it cannot be concluded that younger event attendees will be more likely to be satisfied with the application and become loyal contributors of UGC content.

### H3

Individuals familiar with smartphones are more likely to be more satisfied with the app and become loyal contributors.

To explore this hypothesis, a statistical analysis was conducted to identify the effect of participants’ confidence in using mobile apps on user satisfaction and loyalty. Analysis did not reveal a statistically important effect, and therefore it can be concluded that expertise in the usage of mobile devices is not a differentiating factor. This was an expected finding, since familiarity with mobile devices has increased^3^ and the majority of participants felt rather confident about their mobile skills. Therefore, H3 is rejected, which means that it cannot be claimed that individuals who consider themselves more familiar with smartphones will be more likely to be satisfied with the application or contribute content on a more regular basis.

### H4

Individuals who are adept at filming are more likely to be more satisfied with the app and become loyal contributors.

A statistical analysis on the effect of participants’ filming expertise (self-assessed) on the provided user satisfaction and loyalty scores was carried out to explore this hypothesis. The results did not confirm this hypothesis, which is therefore rejected. In brief, it cannot be concluded that individuals who believe that they are skillful in filming are more likely than others to be more satisfied with the application or to be loyal content contributors.

### H5

Individuals who regularly share content in social media are more likely to be more satisfied with the app and become loyal contributors.

This hypothesis was explored by analyzing the effect of participants’ sharing attitude on their user satisfaction or loyalty scores. In particular, the effect of two sharing attitude indicators was studied, namely sharing videos from events in social media and sharing videos with oneself in social media. None of the explored variables yielded statistically significant effect on SUS or NPS scores, and therefore it can be concluded that contributors’ sharing attitude in social media has no effect on their satisfaction or loyalty, thus rejecting H5. A potential reason is that in the context of this study, contributors did not share in social media in general; instead they knew that they submitted their footage in a closed platform, where the material would be reviewed by professionals.

Overall, findings from the current study suggest that satisfaction and loyalty of potential content contributors is not determined by their age, gender, expertise with mobile apps, filming expertise, or sharing attitudes in social media.

A factor that seemed to influence satisfaction but mostly loyalty was how confident participants felt in their filming in public that day. This was observed in cultural events (festivals), where attendees had perhaps higher self-expectations. At the same time, attendees who reported low confidence in their filming had also reported none to very limited filming expertise in general. Therefore, it can be concluded that it is not the technological medium that has an impact on contributors’ satisfaction and loyalty, but their own filming expertise and self-confidence. In this respect, applications aimed at eliciting user generated videos should make sure to assist inexperienced contributors, for example by providing filming instructions and tutorials.

An important objective factor that substantially affected user satisfaction and loyalty was Wi-Fi / 4G connectivity. It is worth noting that the slow connections were likely to have been the result of overused networks at the events. However, contributors felt frustrated by slow upload speeds and in some cases perceived this slow response as a system bug (as they reported in their responses to open-ended questions). Hence, it is noteworthy that although participants did not report any major usability problems with the application, in the cases with limited connectivity, their perception was that the application was not behaving appropriately and rated the application itself badly. Taking this observation into account, it is crucial to ensure adequate coverage of events which ask for UGC contributions. Currently, 5G networks partially address this need; however there are still open challenges with regard to dedicated bandwidth allocation and resolution of race conditions pertaining to quality of service, such as high bandwidth, low latency, etc. [4]. Furthermore, applications should feature an “upload later” feature to facilitate contributors in uploading the footage at their own convenience. This feature was already included as an option in the evaluated mobile application, however contributors did not use it, as they were instructed to upload footage on site, since the main goal of the evaluation was to assess the on-site experience.

Finally, a side observation pertaining to the evaluation results analysis refers to the importance of combining statistical analysis with qualitative analysis of data. To achieve this, studies can be enriched through combining closed-type questionnaires, with other methods that can elicit qualitative feedback (*e.g.* observations, interviews, open-ended questionnaires). In our case, statistical analysis highlighted important differences, however crucial insights into the causes that actually affected the observed differences were mainly derived from analyzing user responses to open-ended questions.

## Conclusions

Motivated by the vision of enriching the conventional broadcasting experiences with high quality, user-sourced recordings, incorporated in professional productions, this paper has presented a mobile application addressing event attendees and enabling them to record and upload live footage from the event. Furthermore, in order to explore the factors that would make such an endeavor successful, this paper has also presented a study exploring the factors that may actually impact the satisfaction and loyalty of content contributors. In this respect, a field study was conducted with 38 attendees of live sports and festival events in two different countries, who used the described mobile application to record and upload footage. It is noted that prior to field trials the application went through several rounds of evaluation iterations, in order to ensure the highest possible usability, thus allowing the study to focus on factors pertaining to the individual and the overall context.

The results of the study highlight that event attendees are all candidate contributors irrespectively of their demographics (*i.e.* age, gender, technology expertise). This finding is somewhat contradictory to current perceptions that younger attendees might be keener on sharing their on-site experiences. It suggests that everyone could be actively involved in UGC-based efforts, which will eventually increase the received footage pluralism and quantity. Two factors that were crucial for satisfaction and loyalty were filming expertise and Wi-Fi/4G connectivity. In this respect, applications that aim to elicit user videos from live events should be not only easy to use, but also assistive towards contributors, guiding them on how to capture better footage. In addition, footage uploading should be permitted to be carried out at a time that would be convenient for the contributor. This will allow event attendees to focus on actually experiencing the events, rather than mainly interacting with their mobile devices, striving to upload content and becoming frustrated.

Future work should explore additional use cases (*e.g.* other event types), extending existing knowledge and potentially exploring other influential factors. Future studies should replicate best practices that turned out to be of paramount importance for the current study: (i) carry out field studies in live events, which have the potential to reveal contextual factors that cannot be simulated in the laboratory; (ii) select an appropriate combination of evaluation methods (*i.e.* standardized questionnaires and qualitative feedback methods); (iii) pursue international studies, providing the advantage of cultural diversity and promoting wider uptake of the results.

## Footnotes

1 https://www.bbc.co.uk/newsround/49752723.

2 https://www.bbc.com/news/technology-36672001.

3 https://www.nielsen.com/us/en/insights/article/2016/millennials-are-top-smartphone-users/
